# Cooperation between neurovascular dysfunction and Aβ in Alzheimer’s disease

**DOI:** 10.3389/fnmol.2023.1227493

**Published:** 2023-08-16

**Authors:** Niya Wang, Xiang Yang, Zhong Zhao, Da Liu, Xiaoyan Wang, Hao Tang, Chuyu Zhong, Xinzhang Chen, Wenli Chen, Qiang Meng

**Affiliations:** ^1^Department of Neurology, The First People’s Hospital of Yunnan Province, Kunming, China; ^2^The Affiliated Hospital of Kunming University of Science and Technology, Kunming, China; ^3^Institutes of Biomedical Sciences, Shanghai Medical College, Fudan University, Shanghai, China

**Keywords:** Alzheimer’s disease, neurovascular unit, β-Amyloid, blood–brain barrier, cerebral blood flow

## Abstract

The amyloid-β (Aβ) hypothesis was once believed to represent the pathogenic process of Alzheimer’s disease (AD). However, with the failure of clinical drug development and the increasing understanding of the disease, the Aβ hypothesis has been challenged. Numerous recent investigations have demonstrated that the vascular system plays a significant role in the course of AD, with vascular damage occurring prior to the deposition of Aβ and neurofibrillary tangles (NFTs). The question of how Aβ relates to neurovascular function and which is the trigger for AD has recently come into sharp focus. In this review, we outline the various vascular dysfunctions associated with AD, including changes in vascular hemodynamics, vascular cell function, vascular coverage, and blood–brain barrier (BBB) permeability. We reviewed the most recent findings about the complicated Aβ-neurovascular unit (NVU) interaction and highlighted its vital importance to understanding disease pathophysiology. Vascular defects may lead to Aβ deposition, neurotoxicity, glial cell activation, and metabolic dysfunction; In contrast, Aβ and oxidative stress can aggravate vascular damage, forming a vicious cycle loop.

## 1. Introduction

Alzheimer’s disease (AD) is a neurodegenerative brain disease. The most common clinical symptom is progressive memory impairment, along with changes in temperament and behavior, and loss of self-care ability. Amyloid-β (Aβ) protein deposition and neurofibrillary tangles (NFTs) are the two main pathologic hallmarks within the brain (Alzheimer, 1907). The Aβ hypothesis is the earliest and most traditional pathological hypothesis for AD (Hardy and Selkoe, 2002; Selkoe and Hardy, 2016; Hampel et al., 2021). However, numerous clinical trials aimed at reducing Aβ did not significantly change clinical symptoms or the course of the disease, and plaque removal alone was not enough to definitively improve brain performance and enhance cognitive ability, nor was it enough to slow the progression of AD (Evin and Barakat, 2014; Panza et al., 2019; Haass and Selkoe, 2022).

Growing attention has been given to the role played by vascular factors in the pathological mechanisms of AD. Diabetes and hypertension increase the risk for AD (Silva et al., 2019; Cortes-Canteli and Iadecola, 2020; Abdulrahman et al., 2022). In early AD, impaired vascular function has been noted (Iadecola, 2004; Toledo et al., 2013; Sweeney et al., 2019). Our previous study also found that in naturally aging rats, alterations in spatial cognition are preceded by degradation of the hippocampal NVU (Wang N. et al., 2022). Another study in APP/PS1 mice of different ages found that capillary hypofunction preceded Aβ deposition and memory impairment (Wang et al., 2021). Age-related vascular alterations occur concurrently with or even before the pathology of AD, suggesting that they may have a pathogenic role. The two-hit vascular hypothesis for AD, put forth by Zlokovic BV, contends that vascular risk factors (hit 1) result in disruption of the blood–brain barrier (BBB) and decreased cerebral blood flow (CBF), which set off a series of events that precede dementia. Early neuronal dysfunction is caused by the buildup of toxins and capillary hypoperfusion, both of which act independently of the Aβ pathway. Additionally, vascular injury increases Aβ production and decreases Aβ clearance at the BBB, resulting in Aβ accumulation. A rise in Aβ (hit 2) enhances neuronal dysfunction, hastens dementia and neurodegeneration, and aids in the disease’s self-transmission. Tau hyperphosphorylation (p-tau) caused by Aβ protein or hypoperfusion can result in NFTs development (Zlokovic, 2011). The question of how Aβ relate to neurovascular function and which is the trigger for AD has recently come into sharp focus. In this review, we discuss neurovascular changes in AD and their interaction with Aβ.

## 2. Amyloid hypothesis

The Aβ hypothesis was proposed by Hardy and Higgins (1992). They claimed that the primary pathogenic element of AD is the Aβ protein, which is the predominant component of senile plaques. Human genetic investigations have demonstrated that mutations in amyloid precursor protein (APP), presenilin 1 (PSEN1), or presenilin 2 (PSEN2) genes – all of which are involved in the generation of Aβ, are the cause of autosomal dominant familial Alzheimer’s disease (FAD) (Selkoe and Hardy, 2016; Jansen et al., 2019). The APP gene is located on an extra copy of chromosome 21 in about two-thirds of Down syndrome patients who go on to develop AD (Hardy and Higgins, 1992; Lott and Head, 2019). APP is a type I transmembrane protein, that is transported through secretion and endocytic pathways. Under physiological conditions, APP is related to cell adhesion and nutritional support, cell growth, neural differentiation, and synaptic function (Wilkins and Swerdlow, 2017; Zhou et al., 2022). Rice et al. (2019) found that APP regulate neuronal signaling by binding to a variant of gamma-amino-butanoic acid subtype B receptor -GABABR1a. The processing and cleavage of APP depends on different secretases (α, β, γ). The released products can be classified into amyloid and non-amyloid routes depending on which enzymes are used to cleave them. In the amyloid cleavage process, APP is split into two fragments: the N-terminal fragment (sAPP), and the C-terminal fragment. The transmembrane portion of the C-terminal fragment is then hydrolyzed by β-secretase, releasing an Aβ peptide of 39–43 amino acids. The two most common isoforms are Aβ40 and Aβ42 (Annaert et al., 1999; O'brien and Wong, 2011; Selkoe and Hardy, 2016). Aβ production and secretion are driven by synaptic activity, which is the most unique yet common function of the nervous system (Cirrito et al., 2005; Tampellini and Gouras, 2011). Therefore, the production of a small amount of Aβ peptide itself is not toxic, and may even have a physiological function, while the imbalance of Aβ production and clearance and aggregation into oligomers, fibers, plaques are abnormal pathological lesions.

Both Aβ oligomers and fibrils are toxic and can cause tau aggregation, glial activation, inflammatory responses, and neuronal and vascular damage. Studies have found that hybridization between hAPP transgenic mice and hTau transgenic mice can significantly enhance tau deposition and have no effect on Aβ deposition (Roberson et al., 2007). Human Aβ42 oligomers induce tau hyperphosphorylation at AD-associated epitopes and neuro dystrophy in cultured rat neurons, which are prevented by the addition of Aβ antibodies(Jin et al., 2011). Aβ oligomers simultaneously impair synaptic structure and plasticity (Shankar et al., 2008; Sanderson et al., 2021). Oligomeric Aβ accumulation inhibits excitatory junction transmission, but conjointly triggers abnormal patterns of neural circuit activity and epileptic discharges at the network level. Aβ-mediated repressive neural pathology may increase synchrony between excitatory cells and lead to neural network instability (Palop and Mucke, 2010; Zott et al., 2019). Aβ activates microglia and astrocytes, and overactivation of microglia and astrocytes produces a flood of inflammatory cytokines, which in turn cause other types of cell damage (Cuello, 2011; Nordengen et al., 2019).

Previous drugs targeting Aβ have failed to halt the progression of AD. Recently the US Food and Drug Administration (FDA) approved anti-Aβ antibodies: Lecanemab and Aducanumab, which have been shown to mediate the clearance of Aβ plaques in the brain. However, whether addressing Aβ deposition could cure Alzheimer’s is yet to be tested.

## 3. Neurovascular unit dysfunction in AD

The National Institute of Neurological Disorders and Stroke’s 2001 Stroke Progression Conference codified the idea of the NVU, emphasizing the tight connection between the brain and its blood arteries. The NVU is mostly made up of neurons, glial cells (including astrocytes, microglia, and oligodendrocytes) and vascular cells (including endothelial cells, pericytes, or vascular smooth muscle cells (VSMCs)) (Iadecola, 2017). These cells interact together to control CBF and preserve the functionality of the BBB (Zlokovic, 2011; Schaeffer and Iadecola, 2021). Nutrients, oxygen, and energy metabolites are transported to the brain through a network of cerebral arteries, arterioles, and capillaries, and carbon dioxide (CO2) and metabolic waste are transported from the brain to the periphery for removal by cerebral venous reflux (Sweeney et al., 2018; Liu et al., 2019). NVU plays an important role in maintaining brain function and homeostasis.

**Figure 1:**
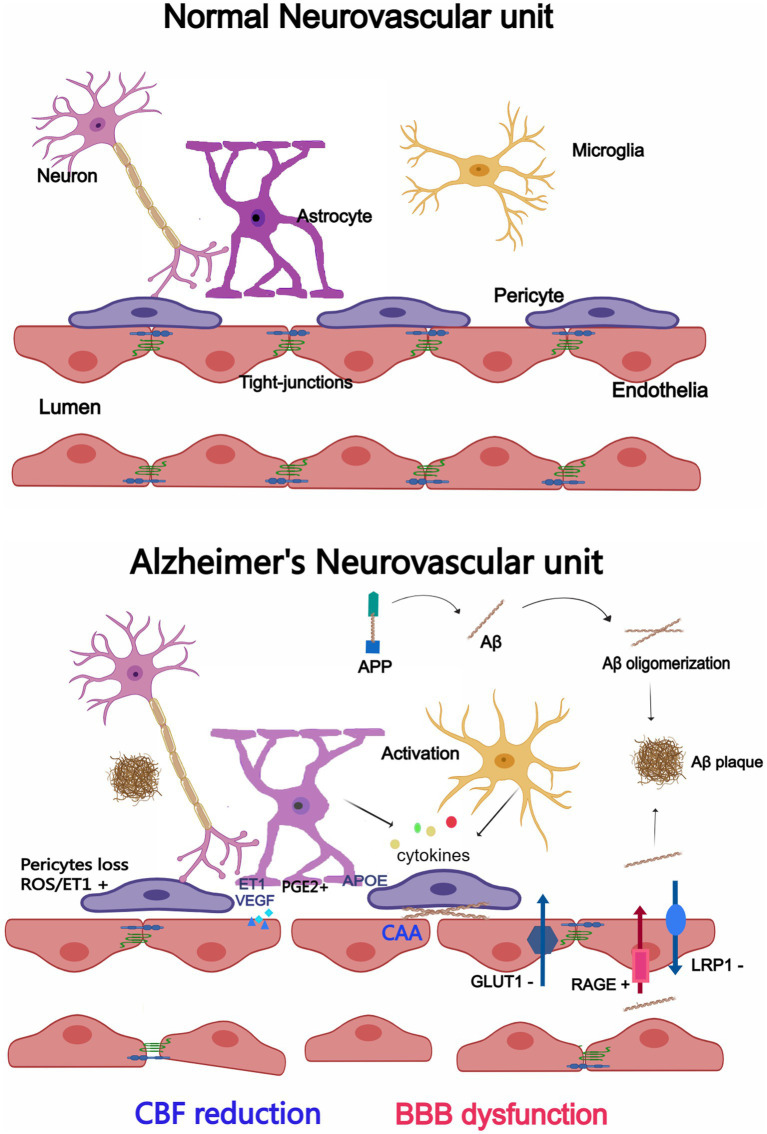
Changes in the neurovascular unit (NVU) between Alzheimer’s disease (AD) and a normal brain. Endothelial cells, pericytes, astrocytes, microglia, and neurons make up the majority of the NVU. The NVU experiences morphological and structural alterations in the AD brain, such as microglia and astrocytes activation and cytokine release, enlarged astrocytic end-feet, pericyte loss, aberrant Aβ transport receptors, and damaged TJs. CBF reduction and BBB dysfunction may result from changes to the NVU. Created with Medpeer.com.

In AD, however, this delicate system is disrupted with significant implications for brain health (Govindpani et al., 2019; Szu and Obenaus, 2021; Yang et al., 2022). Growing evidence suggests a correlation between neurovascular dysfunction and memory deterioration in AD patients. Neurovascular function is critical for information processing, neural connections and synaptic function. Whether neurovascular dysfunction results from or causes AD remains unclear at this time. Currently, changes to the NVU in AD are outlined and summarized as follows, as seen in Figure 1. We will cover them in detail in the following sections.

### 3.1. Pathologic changes of NVU in Alzheimer’s disease

#### 3.1.1. Endothelia

Endothelial cells are the core component of the NVU. They bind together by tight junctions (TJs) that form selective osmotic barriers between the blood and the central nervous system (CNS) and through specific receptors to transport nutrients and remove waste (Bosseboeuf and Raimondi, 2020). Endothelial abnormalities in AD include mitochondrial damage, increased pinocytic vesicles and abnormal receptors (Baloyannis and Baloyannis, 2012; Bosseboeuf and Raimondi, 2020). Dysfunctional mitochondria release danger-associated molecular patterns were found in cerebral endothelial cells in AD, including: loss of mitochondrial membrane potential, increased production of mitochondrial reactive oxygen species (ROS), and permeability of mitochondrial membrane lead to the release of cytochrome C and mitochondrial DNA (mtDNA) into the cytoplasm of endothelial cells (Parodi-Rullán et al., 2021). Pinocytotic vesicles are thought to be a reserve of endothelial membranes that can be used to repair damaged endothelial cells, the number of pinocytotic vesicles in the cerebral capillary endothelium increased significantly in AD patients (Baloyannis, 2015; Levine et al., 2019). Expression of receptor for advanced glycation end products (RAGE) is upregulated, which is responsible for the transfer of Aβ from the periphery to the brain parenchyma; and the transfer of Aβ from the brain into peripheral clearance is reduced by downregulated expression of the low density lipoprotein receptor associated protein 1 (LRP-1) (Cai et al., 2016; Shinohara et al., 2017; Storck and Pietrzik, 2017; Zhou et al., 2021). The glucose transporters GLUT1 and GLUT3 mediate glucose transport to the brain, and early reduced glucose transport associated with reduced expression of GLUT1 and GLUT3 were found in AD (Winkler et al., 2015; An et al., 2018; Kyrtata et al., 2021). Tarawneh et al. (2022) found that increased vascular endothelial-cadherin was associated with Aβ, tau, neurodegeneration in preclinical AD. According to research by Yamazaki et al., synaptic degeneration is associated with the loss of TJs proteins, which is a prevalent occurrence in AD (Yamazaki et al., 2019).

#### 3.1.2. Pericytes

Pericytes wrap around endothelial cells and control capillary contraction to regulate CBF (Winkler et al., 2014; Dessalles et al., 2021). They play a significant role in controlling angiogenesis, TJs, and inflammation in the endothelium (Fisher, 2009; Attwell et al., 2016). A significant reduction of the pericytes coverage of the capillaries within the cortex and hippocampus has been discovered in AD (Sengillo et al., 2013). The earliest structural alteration during AD progression was found to be a loss in pericellular coverage that was reliant on the Braak phase (Kirabali et al., 2020). In the retina of patients with AD, Shi et al. revealed early pericytes loss and vascular amyloidosis (Shi et al., 2020). Signal transmission between pericytes and endothelial cells is intimately related to platelet-derived growth factor subunit β (PDGF-β) and platelet-derived growth factor receptor (PDGFR). In MCI patients, pericytes were damaged and PDGFRβ levels were increased in cerebrospinal fluid (CSF) (Nation et al., 2019). Another study also found a reduction of pericytes in white matter in AD and vascular dementia patients (Ding et al., 2020). In AD patients, capillaries contract specifically by pericytes, while there is no concomitant change in distal arteriole or venule diameter (Nortley et al., 2019; Fisher et al., 2022). Future studies should consider the function of pericytes in the BBB in AD, as recent studies have incontestably that they are crucial in the regulation of neurovascular function, together with BBB formation and maintenance (Armulik et al., 2010; Alcendor, 2020; Lee et al., 2022; Li P. et al., 2022).

#### 3.1.3. Astrocytes

Astrocytes may control BBB function through astrocyte derived factors, and their end-feet surround the endothelium of capillaries, arterioles, and venules (Obermeier et al., 2013; Zhao et al., 2021). Astrocytes regulate arteriolar tone by regulating end-feet prostaglandin E2 (PGE2) expression (Rosenegger et al., 2015). An increase in astrocyte Ca^2+^ triggers the production of arachidonic acid (AA) and its metabolite PGE2, via the PGE2 receptor EP4R, which acts on VSMCs and pericytes to regulate blood flow (Kisler et al., 2017; Sweeney et al., 2018). The BBB is affected by astrocytes in a bidirectional manner, and they could secrete molecules that increase vascular permeability, such as vascular endothelial growth factor (VEGF), nitric oxide (NO), matrix metalloproteinases (MMPs), Apolipoprotein E (APOE), hypoxia inducible factor-1(HIF-1), and endothelin 1 (ET1). These molecules accelerate the breakdown of the BBB. Instead, astrocytes release inhibitors of BBB disintegration, such as hedgehog (Shh), Angiopoietin-1 (ANG-1), and insulin-like growth factor-1 (IGF-1) (Michinaga and Koyama, 2019; Guérit et al., 2021; Lan et al., 2022). Astrocytes express the potassium channels Kir4.1 and Aquaporin-4 (AQP4), which support BBB function by controlling ion and water balance (Jukkola and Gu, 2015). The disruption of the BBB can be caused by astrocytes that carry the APOE4 gene, according to a recent study (Jackson et al., 2022). Reactive astrocytes, which may be caused by Aβ deposits, have been found in the brains of AD patients and in mouse models of AD. Activated astrocytes showed hypertrophy, thickening of processes, and increased expression of intermediate filament protein, glial fibrillary acidic protein (GFAP), vimentin, nestin, and synemin (Preman et al., 2021). A single cell sequencing study found that there was a population of disease associated astrocytes, that expressed a unique set of genes, including genes involved in endocytosis, the complement cascade, and senescence in an AD mouse model (Habib et al., 2020). In AD, reactive perivascular astrocytes display cytoplasmic vacuolization, atrophy, swelling end-feet, decreased astrocytic coverage around endothelial cells and reduced expression of glutamate and lactate transporters (Price et al., 2021; Nehra et al., 2022). These abnormalities may contribute to BBB dysfunction, however, the exact process is still unknown.

#### 3.1.4. Microglia

The majority of the resident immune cells in the CNS are mononuclear phagocytes known as microglia (Lawson et al., 1990). Microglia, the primary element of the NVU, are crucial for controlling CBF and maintaining BBB functionality (Ronaldson and Davis, 2020; Császár et al., 2022; Huang et al., 2023). There are many activated microglia cells close to Aβ plaques in the brains of AD patients and animal models (Itagaki et al., 1989). These microglial cells can polarize into various pro- or anti-inflammatory (M1) or (M2) phenotypes. Tumor necrosis factor-α (TNF-α), interleukin-1β (IL-1β), interleukin-6 (IL-6), interleukin-12 (IL-12), C-C motif chemokine ligand 2 (CCL-2), C-X-C motif chemokine 10 (CXCL-10), ROS and NO are inflammatory cytokines and chemokines secreted by M1 proinflammatory microglia that cause vascular leakage and BBB dysfunction. M2 anti-inflammatory microglia contribute to BBB repair and protection. Haruwaka et al. discovered that systemic inflammation triggers CCR5-dependent migration of resident microglia into the cerebral vessels, where they initially protect BBB integrity before switching to a reactive phenotype and phagocytosing BBB components to start systemic leakage into the parenchyma and cause generalized neuroinflammation (Haruwaka et al., 2019). Additionally, microglia are involved in controlling CBF. According to certain studies, microglia regulate neurovascular coupling through the P2Y12 receptor (P2Y12R) under physiological circumstances. P2Y12R expression on microglia is greatly decreased in AD, which affects cell communication and the cerebrovascular ability to respond to neuronal activity (Kenkhuis et al., 2022). Another study discovered that MCI patients’ microglia and neurons had much higher levels of the catalytic subunit of NADPH oxidase (NOX)- gp91phox (Bruce-Keller et al., 2010; Ansari and Scheff, 2011), and that ROS produced by NOX are crucial for neurovascular decoupling (Park et al., 2005). Microglia mediated NVU dysfunction mainly through inflammatory factors, and most current studies support the Aβ - microglia activation - NVU dysfunction route. However, it may be that with the aging process, inflammation occurs in the brain, leading to abnormal NVU and thus the pathological generation of AD.

### 3.2. Functional changes in the NVU in Alzheimer’s disease

#### 3.2.1. Reduced cerebral blood flow

People with higher CBF rates are less likely to develop dementia or hippocampal and amygdala atrophy, according to early studies using transcranial Doppler measurements of the middle cerebral artery (Ruitenberg et al., 2005; Austin et al., 2011). There is a reduction in CBF in the posterior dentate gyrus and protrusion in patients with MCI or early AD (Austin et al., 2011; Chen et al., 2011). In older people at high risk for AD, CBF abnormalities develop prior to cognitive decline, brain atrophy, and amyloid buildup (Ruitenberg et al., 2005; Knopman and Roberts, 2010; Steinman et al., 2020). While individuals with MCI systematically showed reduced CBF within the posterior cingulate, the results were less consistent in alternative regions, significantly in the cortex (Duan et al., 2021; Swinford et al., 2022). Data in humans show that capillary hypoperfusion occurs before Aβ deposition. A multifactorial data-driven analysis of more than 7,700 brain images and dozens of plasma and CSF biomarkers suggested that cerebrovascular abnormalities are early pathological events in the development of AD (Iturria-Medina et al., 2016). In the hippocampus of AD mice, Zhang et al. discovered a general decrease in mean vessel diameter, volume fraction, and branch angle as well as irregular morphology (Zhang et al., 2019). Our previous research also found that cerebral blood flow in APP/PS1 mice was significantly reduced by using laser speckle contrast image(Wang et al., 2021). The key genetic risk gene for vascular disease and AD is APOE. In transgenic mice designed to target mouse with human APOE4 gene, decreased CBF and vascular dysfunction were similarly observed, and vascular abnormalities in animals expressing APOE4 occurred before neural and synaptic problems (Bell et al., 2012).

The cause and mechanism of decreased CBF are unclear, and could result from the decline of cholinergic neurons that regulate abnormal neurovascular coupling, leading to decreased CBF (Van Beek and Claassen, 2011). Nortley et al. demonstrated limited capillary flow in AD patients due to capillary constrictions caused by pericytes via the ROS-ET1 pathway by analyzing brain biopsy images of patients (Nortley et al., 2019). The inflammatory response also plays an important role in the regulation of CBF, and the release of inflammatory mediators such as IL1β may also help reduce the occurrence of CBF. Mutation of microglia TREM2 receptor increases the production of inflammatory mediators and leads to a decrease in CBF. The capture of neutrophils in capillaries and the formation of clots may also reduce CBF (Cruz Hernández et al., 2019; Korte et al., 2020). Disease-related structural changes in blood vessels and differences in the anatomy of large blood vessels may be important factors for changes in CBF associated with neurodegeneration. Vascular anomalies such as twisted arterioles, reduced capillary density, and enlarged string vessels may also contribute to decreased CBF, in addition to the disruption of neurovascular connections (Baloyannis and Baloyannis, 2012; Yu et al., 2020; Bracko et al., 2021). Reduced capillary density in transgenic AD mouse models with APP23 and APP/PS1, and decreased capillary density near Aβ plaques in Tg2576 models were found. Several studies have reported impaired neurovascular coupling and abnormal CBF in Tg2576 mouse models, prior to the appearance of Aβ deposition, early in disease progression (Niwa et al., 2002). However, other studies have reported no difference in neurovascular regulation between Tg2576 and age-matched wild-type mice at a young age, with abnormal blood vessel function later in disease progression, when Aβ is deposited along blood vessels. There is no clear conclusion as to whether CBF abnormalities predate and are independent of Aβ deposition or are caused by Aβ, which is still controversial and will be discussed further in the following sections.

Neuropathological alterations and neuronal dysfunction resembling those of AD can be brought on by or made worse by hypoperfusion. A 50% reduction in chronic blood flow will lead to significant cognitive changes, a sustained decrease in human CBF of more than 20% will lead to loss of attention, and a decrease in rat CBF of more than 30% will impair spatial memory (Marshall et al., 2001). A decrease in CBF reduces the activity of the Na/K pump and all the processes that depend on it, such as resting potential maintenance and glutamate uptake, and it also leads to the production of adenosine, which inhibits the release of glutamic acid, which in turn affects the function of neurons (Attwell and Laughlin, 2001; Korte et al., 2020). Memory loss, neural dysfunction, synaptic alterations, and the formation of neurotoxic Aβ oligomers are all caused by carotid artery constriction in rats (Wang et al., 2010). Cerebral ischemia, hypoxia and Aβ deposition affect each other. Hypoperfusion can trigger accelerated deposition of Aβ (Thomas et al., 1996; Sun et al., 2006). In rodents, ischemia also causes to accumulate p-Tau and develop filaments (Gordon-Krajcer et al., 2007; Koike et al., 2010). Many of the obvious pathological changes in AD, including Aβ plaque deposition and persistent low-grade inflammation, can be linked to hypoxia caused by reduced blood flow (Park et al., 2019; Salminen, 2021). Hypoperfusion has an impact on structural and functional alterations in the brain and may provide promising indicators that might be used to detect and diagnose AD in its preclinical stage.

#### 3.2.2. Blood–brain barrier disruption

The CNS is isolated from the blood circulation around it by the BBB, a multicellular structure that is specific to the brain. In addition to serving as a barrier, it also actively controls influx and outflow. The influx and outflow of chemicals and ions through certain receptors can be tightly regulated, delivering nutrients and oxygen, and discharging harmful substances such as metabolic wastes and toxins. The BBB maintains brain homeostasis and enables the normal operation of neurons (Obermeier et al., 2013; Uchida et al., 2023). Both histopathological and brain imaging evidence indicated BBB dysfunction in AD. BBB disruption has frequently been identified using measurements of molecules from plasma or serum in the brain parenchyma. Blood-derived components such as plasma proteins, albumin, and IgG have been found in the microvascular regions of the AD brain connected to senile plaques and cerebral amyloid angiopathy (CAA) (Wisniewski et al., 1997; Kurz et al., 2022). Another study found plasma proteins (including prothrombin) in postmortem cortical tissue from Alzheimer’s patients and that protein leakage was more prevalent in patients with at least one APOE4 allele (Zenaro et al., 2017). A frequent sign of BBB breakdown is an elevated CSF/serum or CSF/plasma albumin ratio, which is present in patients with AD (Lin et al., 2021). High spatial and temporal resolution MRI was utilized by Montagne et al. (2015) to examine BBB permeability in the human brain. They discovered age-dependent BBB leakage in the hippocampus, which may cause cognitive impairment. In addition, vascular permeability increases with age in patients with Alzheimer’s or vascular dementia (Farrall and Wardlaw, 2009).

APOE, a significant cholesterol transporter, aids in the transport of lipids and brain injury repair. In addition to influencing the risk of cardiovascular disease, stroke, and other neurological illnesses, the APOE4 allele is thought to be the most prevalent genetic risk factor for late-onset AD (Belloy et al., 2019). While those with the APOE 2 allele had a lower risk of developing AD, homozygous APOE4 carriers were approximately 15 times more likely to do so (Corder et al., 1993; Bown et al., 2007). APOE4 is closely associated with vascular injury (Mielke et al., 2011), Recent studies have found that APOE4 can directly cause damage to the BBB independent of Aβ and phosphorylated tau protein. People who carry one or two copies of APOE4 have leakage in the hippocampus and parahippocampal gyrus, which is more severe in APOE4 carriers who exhibit mild cognitive decline, these effects precede the atrophy of the hippocampus and parahippocampal gyrus (Montagne et al., 2020). Overexpression of ApoE4 usually causes TJ tightness reduction and BBB integrity (Nishitsuji et al., 2011). APOE4 can induce pericytes damage by activating the CypA-MMP9 pathway, leading to BBB disruption (Montagne et al., 2020). Another study found ApoE4 affects pericytes-mediated basement membrane formation, leading to dysfunction of BBB (Yamazaki et al., 2020). Animal experiments have found that the BBB leaks in APOE4 mice, which may be caused by the abnormal expression of MMP9 caused by APOE4 produced by astrocytes, thus affecting the TJs. Selective elimination of ApoE4 in astrocytes restores the integrity of the BBB (Jackson et al., 2022). APOE also plays A crucial role in the metabolism of Aβ protein. APOE2, APOE3 and APOE4 proteins can directly bind to Aβ to form the APOE /Aβ complex, which can alter Aβ clearance, aggregation and deposition (Kanekiyo et al., 2014). The danger of AD and CAA is increased by the APOE 4 allele. APOE4 is one of the major risk genes for AD, and is one with a long history in vascular disease, ApoE4 is a key protein to reveal the relationship between AD and BBB destruction and is also one of the important targets of AD research.

## 4. Aβ and neurovascular dysfunction: causality or causative interaction?

The two-hit concept of AD evolved from the current neurovascular hypothesis, which combines vascular damage and excessive Aβ buildup. However, it is difficult to address which is the primary priming factor. Researchers have long sought the answer to the question, “What is the earliest pathogenic factor of AD?” However, the causal relationship between these factors is unclear, as will be explored below.

### 4.1. Neurovascular dysfunction contribute abnormal Aβ production and clearance

Neurovascular dysfunction contributes to increased production and reduced clearance of Aβ. Hypoxia or low blood flow leads to the production or increased production of Aβ. Reduced blood flow could exacerbate Aβ pathology by causing β or γ-secretases to become active and cause APP cleavage (Sun et al., 2006). Alexander et al. discovered that in hypoxic and ischemic conditions, Hif-1α transcriptionally upregulates BACE1 and non-transcriptionally activates γ-secretase to generate Aβ (Alexander et al., 2022). A twofold imbalance of Aβ efflux and internal transport-related proteins was observed in the cortical arteries of AD mice in animal model studies that found that chronic cerebral hypoperfusion significantly worsened initial AD pathology (Shang et al., 2019). Austin and Katusic (2020) used endothelial nitric oxide synthase (eNOS) heterozygotic knockout (+/−) mice, demonstrating that increased cerebrovascular Aβ is caused by a partial decrease of endothelial nitric oxide. According to Cao et al. (2019) loss of a disintegrin and metalloprotease with thrombospondin type I motif, member 13 (ADAMTS13) led to greater cognitive decline in APP/PS1 mice by speeding up CAA by blocking BBB-mediated Aβ clearance from the brain.

Aβ is cleared by the BBB, which does this by moving Aβ from the interstitial fluid (ISF) into the blood. LRP1 and P-glycoprotein (P-gp), an ATP-binding box (ABC) transporter also known as ABCB1, are the two transport-clearing proteins that have been the subject of most research (Storck et al., 2022). Many of these receptors, transporters, and vectors fluctuate in aging and disease states, leading to abnormal Aβ clearance (Storck et al., 2018; Yang et al., 2020). Inhibition of P-gp and BCRP damaged the BBB and exacerbated AD pathology in a study using AD mouse models (Abdallah et al., 2021). Alzheimer’s patients exhibit low expression of LRP1 and P-gp, which are important transporters of Aβ across the BBB (Van Gool et al., 2019). Decreased expression leads to decreased Aβ transport from the brain to the periphery. However, the increased RAGE expression increased the transfer of blood Aβ into the brain (Yamazaki and Kanekiyo, 2017; Cockerill et al., 2018).

Hypoperfusion caused by abnormal NVU increases amyloidogenic APP processing and promotes the production of Aβ; meanwhile, abnormal BBB leads to reduced Aβ clearance. This eventually led to the aggregation of Aβ deposits. Rescue of the NVU dysfunction not only improves brain homeostasis and neuronal function, but also reduces Aβ deposition, which may be critical for the development of effective therapeutics.

### 4.2. Aβ drives neurovascular dysfunction

Aβ deposition around cerebral vessels is one of the main causes of vascular dysfunction in AD, also called CAA (Shin et al., 2007; Apátiga-Pérez et al., 2022). CAA is a common comorbidity of AD, and is confirmed at autopsy in 75 to 98% of AD patients, CAA gradually reduces vascular reactivity and increases the risk of cerebral hemorrhage and ischemic brain injury (Cupino and Zabel, 2014). Neuroinflammation, chronic hypoperfusion, ischemia, and bleeding injury are all attributed to the Aβ deposits in the blood vessel wall of AD patients, which results in reduced internal diameter and vessel thickness, CAA induced vascular dysfunction reduces perivascular Aβ clearance, creating a vicious cycle of vascular and parenchymal Aβ accumulation (Corovic et al., 2018; Bourassa et al., 2019; Greenberg et al., 2020). Aβ generation, metabolism, and convective clearance of interstitial fluid by perivascular channels are the key similarities between CAA and AD, although clinically, CAA is thought to be distinct from AD (Charidimou et al., 2017; Greenberg et al., 2020). Take et al. used quantum dot nanoprobes and found that Aβ accumulates around human primary cerebral microvascular endothelial cells, the Aβ aggregates hold the cells firmly to the surface of the plate, eventually inhibiting cell movement and causing cell death (Take et al., 2022). Medin amyloid, a fragment of the protein MFG-E8 also known as lactadherin, was recently shown to directly interact with Aβ to increase its aggregation. Medin may be a therapeutic target for reducing vascular damage and cognitive impairment in AD (Wagner et al., 2022).

The Aβ is associated with endothelia and pericytes loss and dysfunction, leading to the NVU dysfunction (Soto-Rojas et al., 2021). Studies have shown that the loss of pericytes in the brains of AD patients and AD mice is associated with increased Aβ deposition, *In vitro* studies have shown that pericytes survival is decreased and NG2 proteoglycan is lost after exposure to Aβ42 and Aβ42 fibrils (Alcendor, 2020). ROS are produced under the influence of Aβ, which causes vasoconstriction and improves some constrictor responses (Niwa et al., 2000, 2001). Recently, Nortley et al. discovered that Aβ constricts human capillaries by communicating with pericytes through ET1 (Nortley et al., 2019). Li found that Aβ1-40 causes BBB dysfunction via the CD36/PINK1/Parkin pathway in pericytes (Li J. et al., 2022). Another study found that Aβ causes BBB dysfunction through the Wnt/β-catenin pathway in brain endothelial cells (Wang Q. et al., 2022). Aβ40 affects neurovascular regulation via a significant and prolonged increase in intracellular Ca^2+^ through TRPM2 channels in brain endothelial cells (Park et al., 2014). Through oxidative stress pathways, Aβ may negatively impact blood vessels. It encourages brain endothelial cells to produce ROS, while ROS scavengers counteract the effects of Aβ on endothelial dysfunction and functional congestion (Park et al., 2008; Leyane et al., 2022).

Although it does not mediate platelet aggregation, APP selectively mediates platelet adhesion to Aβ and works with Aβ to encourage thrombosis in flow-related situations (Visconte et al., 2018). Another study discovered that Aβ42 causes activation of NOXs and integrin IIb-3, platelet adhesion, and thrombosis (Abubaker et al., 2019). Aβ upregulates the endogenous inhibitor plasminogen inhibitor-1 (PAI-1) causing a decrease in tissue plasminogen activator (tPA), resulting in a blockage of increased blood flow due to nerve activation (Park et al., 2020). Experiments with primary endothelial cells from isolated blood vessels and human microvessels have shown that patient-derived Aβ binds to Na + /K + -ATPase α3 subunit (NAKα3) in endothelial cell vesicles to inhibit vasodilation (Sasahara et al., 2021). By activating factor XII (FXII) and interacting with fibrinogen, Aβ may support inflammatory and thrombogenic processes (García-Mejía et al., 2021). Through the intrinsic clotting pathway, Aβ causes FXI activation, the production of thrombin and fibrin. The slow hormones gravikinin (HMWK) and plasma prokallikin (PPK) are both released molecularly as a result of FXII activation, which also activates plasma PPK. Aβ bind to fibrin in addition to its interaction with FXII, strengthening the clot’s resistance to deterioration (Zamolodchikov and Strickland, 2016).

The majority of studies indicate that Aβ triggers NVU disruption, the possible mechanisms include: the deposition of blood vessel wall leads to CAA, damages endothelial cells and pericytes, activates glial cells to produce inflammation, promotes thrombosis, and eventually leads to abnormal CBF and BBB leakage.

## 5. Conclusion and directions

**Figure 2:**
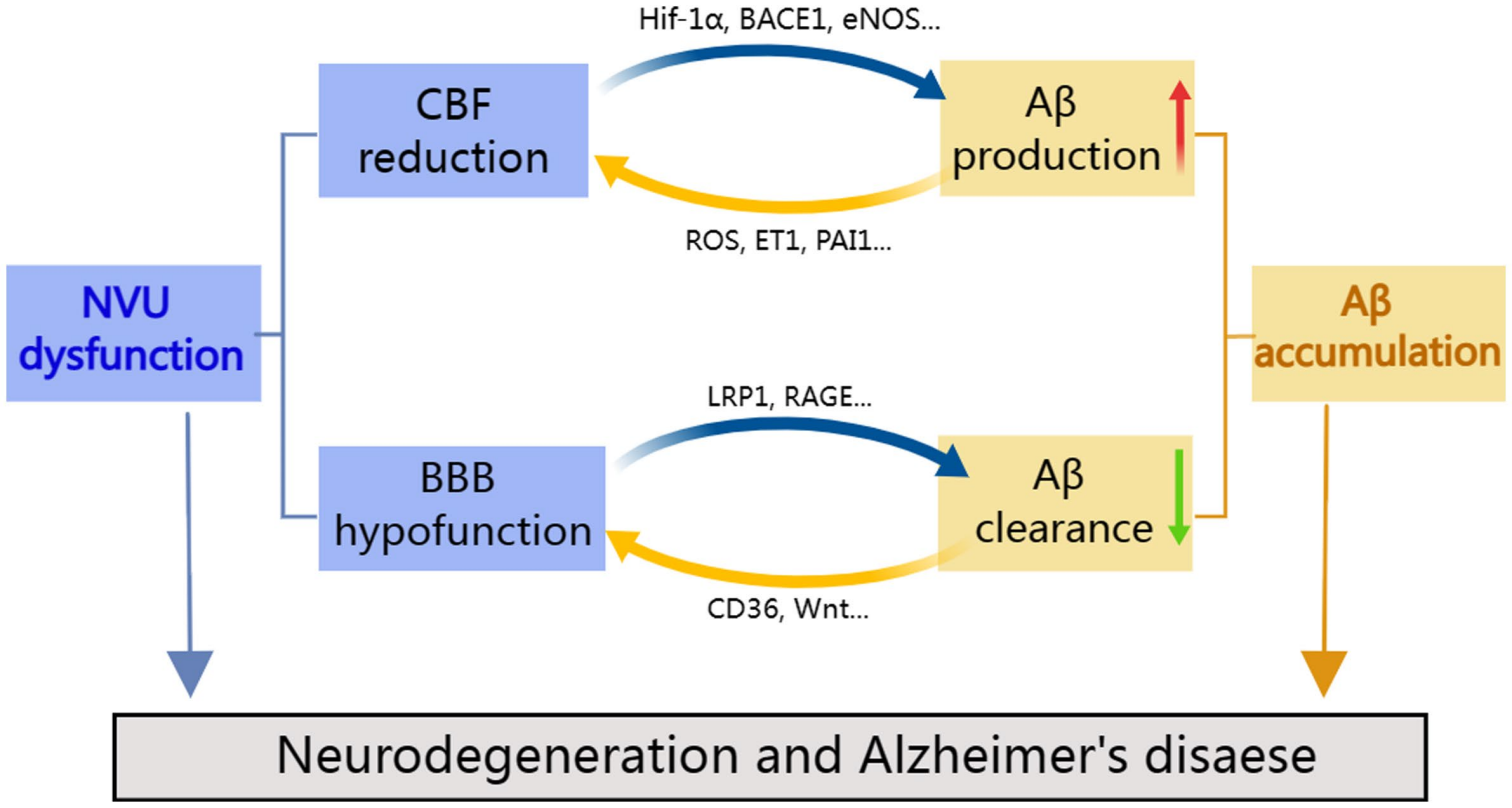
Cooperation between neurovascular dysfunction and Aβ in Alzheimer’s disease. NVU defects may lead to amyloid deposition, in contrast, amyloid toxicity and oxidative stress aggravate vascular damage, resulting in a vicious feedback cycle.

In this review, we provide an overview of the various vascular dysfunctions associated with AD, including changes in vascular hemodynamics, vascular cell function, vascular coverage, and BBB permeability. These vascular defects may contribute to Aβ deposition, neurotoxicity, glial activation, and metabolic dysfunction. Instead, vascular damage is made worse by Aβ toxicity and oxidative stress, creating a vicious cycle loop (Figure 2). Thus, a deeper comprehension of the significance of vascular dysfunction in AD may open up new directions for investigation and therapy. We reviewed the most recent findings about the complicated Aβ-NVU interaction and highlighted its vital importance to understanding disease pathophysiology, drawing on comprehensive human and disease model data. Given the relationship between Aβ and neurovascular changes, it is possible to comprehend why early prevention of vascular risk factors in the elderly population may be a successful strategy for the prevention of AD and why current investigational drugs targeting Aβ clearance do not work well. Precision medicine strategies for the early diagnosis, treatment, and prevention of AD may have a physiologically informed aim in cerebrovascular interactions with Aβ and structural brain pathology.

## Author contributions

NW and QM contributed to the conception of the study. NW and XY wrote the manuscript and drew figures. ZZ, DL, WC, and QM corrected the manuscript. XW, HT, XC, and CZ contributed to search and organize literatures. All authors contributed to the article and approved the submitted version.

## Funding

This research was supported by the Department of Science and Technology Program of Yunnan Province (Grant numbers: 202301AT070039 and 202301AY070001-233), Yunnan Province First People’s Hospital – Kunming University of Science and Technology joint project (KUST-KH2022012Y), and The Yunnan Municipal Health Commission and First People’s Hospital of Yunnan Province (Grant numbers: 2022-KHRCBZ-B04 and KHBS-2022-017).

## Conflict of interest

The authors declare that the research was conducted in the absence of any commercial or financial relationships that could be construed as a potential conflict of interest.

## Publisher’s note

All claims expressed in this article are solely those of the authors and do not necessarily represent those of their affiliated organizations, or those of the publisher, the editors and the reviewers. Any product that may be evaluated in this article, or claim that may be made by its manufacturer, is not guaranteed or endorsed by the publisher.
